# Advanced Optoelectronic Applications of Nanopillar Arrays Fabricated by Glancing Angle Deposition

**DOI:** 10.3390/nano15201555

**Published:** 2025-10-13

**Authors:** Yating Fang, Lin Yang, Zhifeng Huang

**Affiliations:** 1 Department of Chemistry, The Chinese University of Hong Kong (CUHK), Shatin, N.T., Hong Kong SAR, China; fangyating@link.cuhk.edu.hk; 2 Shenzhen Research Institute, The Chinese University of Hongkong, No. 10, 2nd Yuexing Road, Nanshan, Shenzhen 518057, China

**Keywords:** glancing angle deposition, nanopillar arrays, optoelectronics, photovoltaics, photodetection, light emission diodes

## Abstract

Glancing angle deposition (GLAD) is a unique physical vapor deposition technique to enable wafer-scale production of close-packed nanopillar arrays (NaPAs) made of a wide range of inorganic and organic materials and engineerable structures, offering great potential for advanced optoelectronic applications. By flexibly controlling substrate rotation during GLAD, this technique enables intricate sculpture of nanopillars in vertical/tilted column, helix, zigzag, and square spiral shapes or a combination of these shapes along the vertical growth axis. In particular, NaPAs exhibit unique engineerability in their material/structure-determined optical, electronic, chemical, mechanical, and morphological properties, making them versatile for significant applications in photovoltaics, photodetection, photocatalysis, and advanced displaying. In this review, we provide a comprehensive overview of recent advancements in optoelectronic applications of GLAD-fabricated NaPAs by exploring the relationship between structural features and device functionality. Additionally, we discuss the technical challenges associated with GLAD, such as scalability, material compatibility, and fabrication precision, and address prospects to produce next-generation optoelectronic devices.

## 1. Introduction

Rapid advances in materials science are continuously redefining the frontiers of optoelectronic technologies. Optoelectronics plays a pivotal role in technologies ranging from solar cells [1,2,3,4] and photodetectors [5,6,7] to light emission diodes (LED) [8,9] and communication systems [10]. The performance of these devices essentially depends on the development of materials that combine excellent electrical conductivity with strong light–matter interaction, enabling next-generation, energy-efficient, high-speed optoelectronic technologies.

Since nanomaterials have been widely investigated due to their unique properties emerging at the nanoscale, they have gained significant attention to revolutionize optoelectronic devices [11,12,13]. Given their engineerable structures, high surface area, and tunable optical and electrical properties, nanomaterials offer exciting chances to improve optoelectronic performance [14,15,16].

Nano-fabrication techniques can be generally divided into two categories [17], i.e., wet-chemical methods [18] and vapor-phase deposition approaches [19,20]. The former methods, including sol–gel processing [21], hydrothermal growth [22], electrochemical deposition [23,24], and solution-based film techniques (such as spin coating [25], spray coating [26], and dip coating [27]) have been widely used due to their simplicity and low cost, especially for laboratory-scale fabrication. However, these methods usually suffer from the limitations of requiring precise structural control, high production repeatability, and scalability. On the contrary, other techniques (such as chemical vapor deposition [28], physical vapor deposition (PVD) [29], and atomic layer deposition (ALD) [30]) offer controllable fabrication in terms of film thickness, stoichiometry, and crystalline orientation, and enable conformal growth of nano-films on diverse substrates [31]. Among these technologies, glancing angle deposition (GLAD, a unique PVD) stands out as a versatile technique to reliably form nanostructures with tailorable geometries, properties, and functions in large areas. Given that pursuing high efficiency, spectral tunability, and multifunctionality in optoelectronic devices continues to drive demand for precise control of materials at the nanoscale, GLAD paves the way for bottom-up mass-production of anisotropic, ordered nanostructures with tunable morphology, surface area, and crystallographic orientation.

This review provides a comprehensive overview of the advantages of GLAD and its application in fabricating nanomaterials for advancing optoelectronic devices. First of all, it introduces the fundamental principles of GLAD and discusses nanomaterials and nanostructures commonly used in optoelectronic devices, whereby both the strengths and limitations of GLAD to create nanostructured thin films are highlighted. Then, recent progresses are summarized across key optoelectronic applications, including photovoltaics, photodetection of non-polarized and polarized light in the visible and near-infrared region, photocatalysts, and LEDs. In addition, challenges related to scalability, reproducibility, and integration with other existing technologies are examined and perspectives are discussed. Overall, this review aims to offer significant insights into how GLAD can advance optoelectronic device performance and stimulate further innovations in related fields.

## 2. GLAD of Nanopillar Arrays

**Figure 1:**
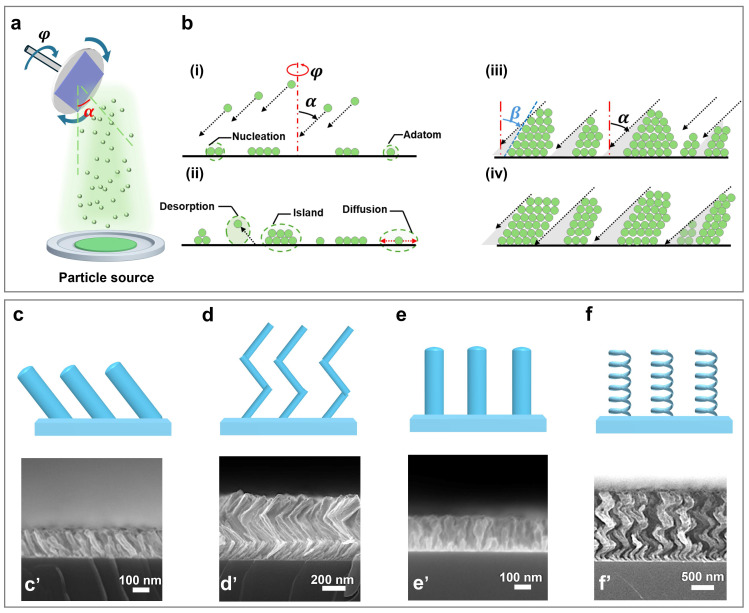
Glancing angle deposition (GLAD) of nanopillar arrays (NaPAs), with flexible engineering of the pillar shape. (**a**) Schematics of GLAD: materials are evaporated to condense on a substrate along the deposition angle α in terms of the normal direction of the substrate, while the substrate can be rotated with respect to the azimuthal angle φ. (**b**) Schematics of forming tilted nanopillars during GLAD: (**i**) adatom adhesion and nucleation on the substrate; (**ii**) possible desorption, island formation, and surface diffusion of adatoms; (**iii**,**iv**) α-induced self-shadowing effect. (**c**–**f**) Schematics; (**c’**–**f’**) scanning electron microscopy (SEM) cross-sectional images of the GLAD-fabricated nanopillars in a shape of (**c**,**c’**) tilted column; (**d**,**d’**) zigzag; (**e,e’**) vertical column; (**f**,**f’**) helical. This figure was created by the authors.

GLAD is a unique PVD technique wherein a material flux is evaporated and directed at a glancing deposition angle (α) with respect to the normal direction of a substrate [32] where evaporated materials condense (Figure 1a) [33]. Upon arrival at the substrate, adatoms go through dynamic processes, including adsorption at available sites, possible desorption back into the vapor phase, and surface diffusion across adjacent adsorption sites driven by thermal energy and local kinetic interactions to reduce surface free energy. These mobile adatoms may aggregate to form stable nuclei or small islands, which subsequently act as preferential sites for further growth. As deposition proceeds, the oblique incident flux (α) induces self-shadowing; meanwhile, surface diffusion modulates the column morphology, ultimately leading to the formation of tilted nanopillars (Figure 1b,c,c’) [34]. The anisotropic growth makes the as-deposited NaPAs inclined toward the vapor source, with the tilting angle β determined by α, as given by Tait et al. [35].

(1) tanα=2tanβ

(2) $$\beta =\alpha -arc\sin\frac{1-\cos\alpha }{2}$$

The existence of these two equations reflects the complexity of the deposition process where both α and β are expressed in radians. Equation (1) is generally applicable to the deposition of low-diffusivity materials and/or at low substrate temperature, while Equation (2) is more suitable when surface diffusion contributes significantly to the growth dynamics [36]. Their applicability thus depends on material type, substrate temperature, deposition rate, and vacuum level of GLAD.

When substrate movement is introduced via control of φ during GLAD, the as-deposited nanostructures can be sculpted in various shapes. For instance, when a substrate is alternately switched back and forth, a zigzag NaPA will be created (Figure 1d,d’) [37,38]. Unidirectional fast rotation of a substrate enables one to generate columnar NaPAs (Figure 1e,e’) [39], while unidirectional slow rotation results in the formation of helical NaPAs (Figure 1f,f’) [40,41]. Specifically, deposition at a glancing angle with fast substrate rotation, consistent with Figure 1c,c’, but in a short deposition period results in the growth of nanoparticles (NPs) [42]. The substrate movement dynamically modulates the shadowing profile to induce tunable morphology, growth orientation, and porosity in NaPAs, providing a library of nanomaterials and nanostructures with tailorable optical, electrical, and mechanical properties for performing advanced optoelectronic functions.

Furthermore, by controlling a set of deposition parameters (including α, substrate rotation rate, deposition rate, substrate temperature, vacuum level, and deposition duration), the growth direction, size, and packing density of NaPAs can be precisely tuned [43], while maintaining a stable vacuum is essential to achieve reliable engineering of nanostructures. Notably, introducing oxygen or other background gases during GLAD can disrupt the vacuum conditions and significantly affect the morphology, stoichiometric coefficient, and defects of NaPAs [44]. Table 1 summarizes a wide range of inorganic and organic materials successfully sculpted to form NaPAs through GLAD for optoelectronic applications, and the material scope can be further extended via co-deposition [45], layer-by-layer deposition [46,47,48], post-deposition treatments [49,50], and conformal coating processes [51].

## 3. Fundamental Functions of Nanopillar Arrays in Diverse Optoelectronic Devices

GLAD uniquely enables precise control over nano-morphologies, compositional stoichiometry, chemical valence states, and crystallinity. Such high level of tunability facilitates the design of nanomaterials tailored for a wide range of optoelectronic applications. For instance, antireflection (AR) columnar NaPAs can be applied to enhance light trapping [39], while vertically protruding NaPAs tend to improve charge transport, facilitate charge collection, and strengthen surface interactions [102]. Among the diverse structures produced by GLAD, helical NaPAs are of particular significance due to their chiroptical properties, enabling the selective emission and detection of left-handed or right-handed circularly polarized light (CPL). Furthermore, helical NaPAs exhibit superior mechanical flexibility, as their geometries help relieve interfacial stress and minimize residual strain during bending or mechanical deformation. Zigzag NaPAs and nanotree structures offer increased surface area and more active sites, thereby promoting interfacial reactions and enhancing catalytic efficiency in photochemical applications [103,104,105]. Noble metal NPs prepared via GLAD exhibited strong localized surface plasmon resonance (LSPR) which significantly improved light absorption and concentrated electromagnetic fields, making them promising candidates as highly sensitive photodetectors and efficient photocatalysts [106,107].

**Figure 2:**
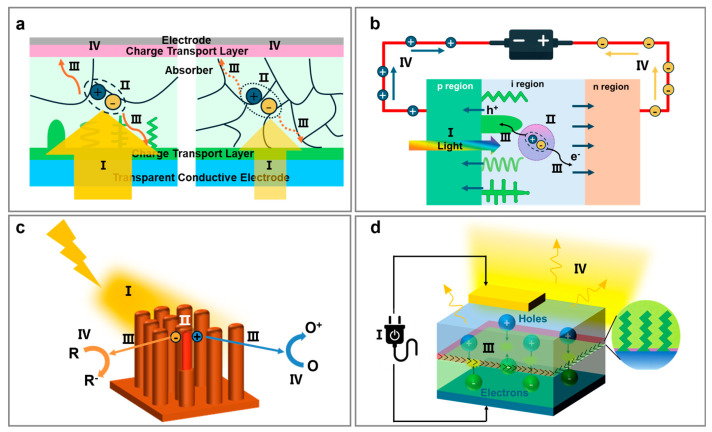
Schematics of the dynamics of photo-induced charge carriers in nanopillar array (NaPA)-enhanced optoelectronic devices, including (**a**) perovskite solar cells; (**b**) photodetectors; (**c**) photocatalytic systems; (**d**) light-emitting diodes (LEDs). Step I: (**a**–**c**) light harvesting, (**d**) charge injection. Step II: generation of excitons (or electron–hole pairs), which are absent in (**d**). Step III: (**a**–**c**) separation of excitons and charge transport via internal electric fields or built-in electric field, (**d**) charge transport stimulated by external electric biases. Step IV: (**a**,**b**) charge extraction, (**c**) surface redox reactions, (**d**) photon emissions via radiative recombination of electrons and holes. This figure was created by the authors.

Given their nanostructure-determined properties, researchers have recently explored the applications of GLAD-fabricated NaPAs across various optoelectronic devices, including solar cells, photodetectors, photocatalysts, and LEDs. The specific roles of NaPAs in these devices are depicted in Figure 2. In terms of photovoltaics and photodetectors (Figure 2a,b), NaPAs sculpted in helical, vertical column, and zigzag shapes primarily enhance light absorption (Step I), exciton separation (Step II), and charge transport (Step III) [108,109]. Their anisotropic morphology and high aspect ratio are beneficial to improve antireflection performance and light harvesting in photovoltaic-active absorbers, increasing the efficiency of exciton generation efficiency. Furthermore, vertically aligned NaPAs act as directional pathways for charge transport, shortening carrier diffusion lengths and mitigating recombination losses at grain boundaries. In this way, NaPAs simultaneously address the optical and electronic challenges, enabling higher external quantum efficiency in both solar cells and photodetectors.

For photocatalytic and photoelectrochemical catalysis, NaPAs make a similar contribution to enhance optoelectronic performances (Figure 2c). The NaPAs facilitate light absorption, exciton generation and separation, and charge transport to the NaPAs’ surfaces (Step I–III), while the large surface area of the NaPAs provides abundant active sites for surface redox reactions (Step IV). More importantly, the porous and ordered NaPAs allow for multi-electron transfer processes and enhanced reactant diffusion, making them particularly effective for driving surface catalytic transformations that go beyond merely charge extraction. Thus, in contrast to solar cells and photodetectors, wherein NaPAs mainly act as optical or electrical enhancers [110], in photocatalysis they additionally serve as photocatalysts [111].

In LEDs (Figure 2d), where carrier injection (Step I) rather than photon absorption initiates the process, NaPAs primarily support charge transport (Step III) and irradiative recombination of charge carriers (Step IV) [112,113]. Their anisotropic nanostructures facilitate current injection and reduce local thermal accumulation, thereby promoting radiative recombination [114,115]. Furthermore, the anisotropic interfaces enhance light outcoupling (Step IV) by reducing Fresnel reflection and suppressing total internal reflection [116,117,118]. These contributions are particularly important for high-power and flexible LEDs, where conventional planar designs suffer from poor thermal dissipation and limited extraction efficiency.

Overall, GLAD-fabricated NaPAs provide a structural strategy to mitigate photon management (Step I/IV), carrier dynamics (Step II/III), and interfacial reactions (Step IV) across diverse optoelectronic systems. In photovoltaics and photodetectors, NaPAs primarily enhance light harvesting, exciton separation, and charge transport. In photocatalysis, they promote charge separation and provide structurally defined catalytic scaffolds that expose abundant active sites. In LEDs, they facilitate carrier injection and efficient photon emission. NaPAs highlight how structural engineering can systematically tailor distinct stages of optoelectronic processes across a broad range of devices.

## 4. Applications of Nanopillar Arrays in Photovoltaic Devices

Developing high-efficiency, cost-effective photovoltaic technologies is essential for reducing carbon emissions and enabling sustainable energy transitions. To compete with conventional energy sources, next-generation photovoltaic systems must incorporate advanced strategies to improve power conversion efficiency (PCE).

Fundamentally, the PCE of a solar cell is determined by three key processes, including photon absorption, charge carrier separation, and charge collection [119,120]. A typical thin-film solar cell consists of a light-absorbing active layer sandwiched between an electron transporting layer (ETL) and a hole transporting layer (HTL), with front and back electrodes to extract the generated carriers [121]. To optimize these processes, the integration of effective AR coatings and advanced ETLs and HTLs, especially those featuring functional nanostructures, is crucial [122,123]. Nanostructured design can reduce exciton recombination and improve the formation of Ohmic contacts, thereby enhancing device performance [124,125]. Therefore, nanostructured materials, particularly those fabricated via GLAD, have demonstrated considerable promise in overcoming these challenges [126,127,128]. GLAD enables the fabrication of tailored nanostructures such as vertically aligned and helical NaPAs, which enhance light absorbing and facilitate efficient charge transport [62,129]. Furthermore, absorber layers grown on these nanostructures often possess higher crystallinity and reduced defect densities, contributing directly to improved mechanical and operational stability [130,131]. Long term stability under operational and mechanical stress is equally important for practical applications. Techniques like maximum power point tracking are commonly employed to augment real-time output, while cyclic bending tests have been used to assess mechanical durability, especially for flexible or wearable photovoltaic applications [132,133,134].

The usage of photons from sunlight is mainly determined by the bandgap of the active absorber layer [135,136]. Absorber materials can be broadly categorized into indirect bandgap semiconductors (e.g., crystalline silicon), direct bandgap semiconductors with moderate absorption strength (e.g., GaAs, CIGS), and emerging strong-absorption materials (e.g., organic molecules and halide perovskites). Indirect bandgap materials (e.g., Si) exhibit weak absorption near the band edge and require thick absorber layers to achieve sufficient light absorption [137,138]. In contrast, direct bandgap materials (e.g., GaAs, CIGS) allow efficient absorption in much thinner layers, improving carrier collection and reducing recombination losses [139]. Notably, halide perovskites possess exceptionally high absorption coefficients (~10^5^ cm^−1^), enabling efficient light capture in ultrathin layers which is highly beneficial for device fabrication and integration. Consequently, a broad range of photovoltaic technologies has been developed to convert incident photons into electricity. These include silicon-based solar cells [140], Cu(In,Ga)Se_2_ (CIGS) [141], Cu_2_ZnSn(S,Se)_4_ (CZTSSe) [142], CdTe [143], GaAs [144], and SnS thin-film solar cells [145], dye-sensitized solar cells (DSSCs) [146], organic photovoltaics (OPVs) [147,148], and perovskite solar cells (PSCs) [149]. PSCs have seen rapid development since 2013 owing to their tunable bandgaps, high absorption coefficients, and long carrier diffusion lengths, leading to dramatic improvements in PCE [150]. Given the characteristics outlined above, this review categorizes the photovoltaic technologies into non-perovskite systems (e.g., DSSCs, OPVs, GaAs, SnS, all-oxide, CIGS, CdTe) and PSCs.

### 4.1. Non-Perovskite Solar Cells

Non-perovskite solar cells, including DSSCs, OPVs, and various thin-film technologies (such as GaAs, SnS, CIGS, and CdTe) have benefited from the integration of nanostructures fabricated via GLAD. Although these solar cells differ in their absorber materials and device configurations, the incorporation of aligned NaPAs typically contributes to increased surface area, which is critical for processes such as dye loading in DSSCs, charge separation in OPVs, or improving the active interfaces in thin-film solar cells. The NaPAs offer direct pathways for charge transport, minimizing recombination losses. Moreover, the light management capability of these nanostructures, including scattering, diffraction, and, in some cases, plasmonic enhancement, improves photon absorption, particularly in devices that rely on thin active layers. Finally, the tunability of GLAD processes allows precise control over parameters such as the tilt angle, shape, size, and composition of NaPAs, enabling tailored optimization for desired photovoltaic technologies.

**Figure 3:**
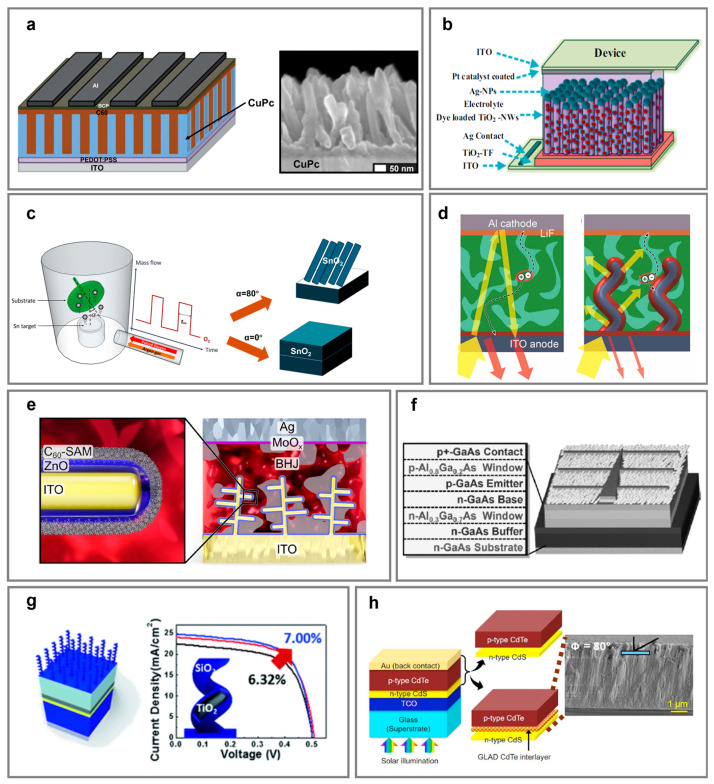
Photovoltaic application of GLAD. (**a**) Schematic illustration and SEM image of vertically aligned CuPc nanopillar arrays (NaPAs) serving as the absorber layer in an organic photovoltaic device. The left panel shows the device architecture, consisting of ITO/PEDOT:PSS/CuPc/C60/BCP/Al from bottom to top. The right panel presents a cross-sectional SEM image of the CuPc NaPAs, with a 50 nm scale bar. (**b**) Schematic of Ag-nanoparticle-coated TiO_2_ photoanode in dye-sensitized solar cell. The device structure consists of ITO/TiO_2_ thin film/dye-loaded TiO_2_ NaPAs/Ag nanoparticles/Pt-coated ITO. (**c**) Schematic of DC reactive magnetron sputtering system employed for the fabrication of SnO*_x_* NaPAs using a combination of GLAD and reactive gas pulsing process techniques. (**d**) Schematic of helical ITO NaPAs as transparent conductive electrodes with integrated antireflection in organic photovoltaics.Yellow arrows indicate incident and absorbed light, red arrows represent reflection losses, and black dashed lines denote charge transport pathways. (**e**) ITO nanotrees coupled with ZnO-C60 in bulk heterojunction organic photovoltaics. The device configuration is ITO/ITO nanotree/ZnO/C60-SAM/PDPPTT-T-TT:PC_71_BM/MoO*_x_*/Ag. (**f**) Schematic of a GaAs solar cell employing ITO NaPAs as the conductive antireflection (AR) coating. The epitaxial structure is illustrated on the left. (**g**) Schematic of TiO_2_@SiO_2_ core@shell NaPAs deposited on CIGS as the AR coating with 10% improvement in PCE. The J–V curves (black: pristine CIGS, red: with TiO_2_, blue: with TiO_2_@SiO_2_) indicates a 10% enhancement of PCE. (**h**) Schematic of CdS/CdTe heterojunction solar cells with and without GLAD-fabricated titled CdTe NaPAs between the wurtzite CdS and zinc-blended CdTe layers and cross-section SEM image of titled CdTe NaPAs. (**a**) Adapted with permission [100]. Copyright 2011, Royal Society of Chemistry. (**b**) Adapted with permission [52]. Copyright 2024, Springer Nature. (**c**) Adapted with permission [80]. Copyright 2024, Elsevier. (**d**) Adapted with permission [73]. Copyright 2014, Wiley. (**e**) Adapted with permission [74]. Copyright 2017, American Chemical Society. (**f**) Adapted with permission [161]. Copyright 2008, Wiley. (**g**) Adapted with permission [61]. Copyright 2019, Royal Society of Chemistry. (**h**) Adapted with permission [83]. Copyright 2020, Elsevier.

The active layer determines photon absorption, exciton generation, and initial charge separation [151]. GLAD-enabled nanostructures contribute by increasing surface area, optimizing molecular orientation, and enhancing light-trapping. In OPVs, columnar copper phthalocyanine (CuPc) NaPAs grown on PEDOT:PSS-coated ITO substrates which optimize the donor layer morphology by tailoring the pillar diameter, spacing, and height were used to enhance small-molecule OPV s’ performance (Figure 3a) [100]. In order to regulate a hybrid organic–inorganic interface, incorporating copper iodide (CuI) into GLAD via a thermal process at 400 °C promotes strong interfacial interaction with zinc phthalocyanine (ZnPc) [152], leading to the formation of vertically columnar ZnPc/CuI NaPAs with an ordered layer-by-layer π-π stacking orientation of ZnPc on the CuI NaPAs. This molecular arrangement enhances light absorption and increases surface roughness, thereby suppressing exciton recombination [153]. OPVs integrated with columnar ZnPc/C60 NaPAs possess an optimized PCE which is about 3-fold larger than that of the planar OPVs. It is proposed that the integration of organic NaPAs is a promising route to fabricate highly efficient small-molecule OPV devices.

Furthermore, a nanostructured ClAlPc donor layer, fabricated by GLAD, was integrated in the ClAlPc:C60 BHJ OPV device [101]. By depositing the top ClAlPc layer at a glancing angle, they achieved an anisotropic rough layer whereby interfacial contact and charge transport was improved due to the built-in field created by bilayer ClAlPc. This optimized ClAlPc:C60 BHJ layer with a thickness of 40 nm achieved a PCE of 1.16%. This design strategy represents a promising approach for improving voltage output in vacuum-processed small-molecule OPVs.

Efficient charge extraction depends on the design of ETLs and HTLs. Herein, GLAD-fabricated NaPAs provide directional pathways and energy level alignment for improved carrier mobility and suppressed recombination. In DSSCs, zigzag TiO_2_ NaPAs were fabricated using electron-beam GLAD at various deposition angles ranging from 53 to 86° [60]. TiO_2_ NaPAs synergistically improved dye loading, electron transport, and light-trapping, with optimal performance at 73°. Furthermore, Ag NP-decorated TiO_2_ NaPA photoanodes were prepared by varying the speed of the substrate rotation using GLAD [52]. The incorporation of Ag NPs into the TiO_2_ NaPA photoanode primarily serves to enhance light absorption via LSPR. Upon light irradiation, electrons in the conduction band of Ag NPs generate a strong localized electromagnetic field at the Ag/NaPA’s interface, which increases the optical path length and light scattering within the photoanode. A slower rotation rate of the substrate yields vertically aligned, compact arrays that enhance dye adsorption and charge transport, while faster rotation may produce more porous nanostructures with reduced surface area and collection efficiency (Figure 3b). Therefore, the TiO_2_ NaPA photoanodes used the GLAD technique to maximize the surface area for dye loading and facilitate direct electron pathways, improving interfacial contact with electrodes, and boosting light-trapping efficiency, collectively contributing to higher DSSC performance.

To improve the performance of eco-friendly SnS thin-film solar cells, Djeffal et al. numerically investigated a novel device structure incorporating GLAD-fabricated SnO_2_ NaPAs as the ETL and a graded-bandgap SnS_1-x_Se_x_ absorber. In Capacitance Simulator-1D simulations, the optimized SnO_2_/graded SnSSe/Mo structure delivered the highest PCE, which more than doubled that of planar CdS/SnS solar cells [78]. This enhanced performance is majorly attributed to the SnO_2_ NaPAs, while the compositional grading in the SnSSe layer broadened the absorption band edge and strengthened the built-in electric field. Furthermore, SnO*_x_* films were evaporated via a direct current (DC) reactive gas pulsing process combined with GLAD (Figure 3c) [80]. By varying the oxygen pulsing time from 0 to 20 s, the bandgap of SnO*_x_* was effectively tuned from 0.9 eV to 3.56 eV. The resulting all-oxide SnS solar cell demonstrated a high PCE of 3.41%. These findings highlight the potential of integrating the GLAD with in situ oxidization processes to finely modulate the structural and electronic characteristics of metal oxide semiconductors via controlled gas-phase dynamics. However, the relatively modest PCE suggests that additional refinements in interfacial energy alignment, defect passivation, and charge carrier transport are still imperative to fully exploit the potential of SnS-based all-oxide solar cells.

Electrodes fabricated by GLAD contribute to effectively enhancing charge separation and managing incident light [154,155]. Their morphology can be tailored to enhance mechanical flexibility, optical transmittance and charge injections or extraction. Indium tin oxide (ITO) NaPA sculpted in helical and tree-like shapes have been developed, offering promising potential for future flexible and transparent applications. For instance, helical ITO NaPAs serve as multifunctional electrodes for bulk heterojunction OPVs to simultaneously improve light absorption and charge transport from the active region to the back electrode (Figure 3d) [73]. During GLAD, the substrate temperature is elevated to approximately 550 °C through halogen lamp heating, enabling partial melting or softening of the ITO adatoms. This localized quasi-liquid phase at the nucleation sites facilitates vapor–liquid–solid axial growth, while the oblique incident vapor flux and azimuthal substrate rotation ensures directional and shadowing control for helical nanostructures [156]. As a result, the optical and electrical improvements enabled by ITO NaPAs result in a 10% increase in short-circuit current density and PCE of OPVs, which is attributed to a strong light-scattering effect caused by the three-dimensional helical NaPA structure. Moreover, more complex ITO nanotrees further improve the electrode surface area and facilitate conformal coatings of functional layers like ZnO followed by the self-assembly of carboxylic acid functional group (COO)^−^-modified C60 on ZnO through the formation of COO^−^ ZnO contact (Figure 3e) [74]. When the height of the ITO nanotrees varied from 0 to 50, 75, 100, and 120 nm, statistically significant variations in photovoltaic performance were observed, with the maximum PCE achieved at a height of 75 nm. Further investigations in light management and mechanical robustness have been conducted through tunable ITO NaPAs [157,158,159].

GLAD-fabricated NaPAs are effective AR coatings due to their graded refractive index and multiple scattering effects, critical for thin-film photovoltaics with limited optical path lengths [160]. The integration of tilted ITO NaPAs deposited on the p-type Al_0.8_Ga_0.2_As window layer as an AR coating in GaAs solar cells yields significant optical benefits (Figure 3f) [161]. Compared to devices without an AR coating, the NaPAs improve transmittance by up to 28%, resulting in nearly 42% PCE enhancement in the generated photocurrent. Analogous to ITO NaPAs, core@shell TiO_2_@SiO_2_ helical NaPAs have also been explored for AR coating, providing gradual refractive index transitions and improved light trapping. When the core@shell TiO_2_@SiO_2_ helical NaPAs were integrated in CIGS solar cells (Figure 3g) [61], the thickness of TiO_2_ core was finely tuned via a finite-difference time–domain simulation, and giving it a thickness of 150 nm maximized the electric field intensity in the spectral region of 400–1100 nm, resulting in a 5.2% boost in current density. Similarly, a conformal 3 nm-thick SiO_2_ shell introduced a gradual refractive index transition to air, further suppressing surface reflection.

Nanostructured interfacial layers grown via GLAD can assist in energy band alignment, defect passivation, and lattice mismatch which are critical for efficient carrier extraction and reduced recombination losses. Vertically oriented NaPAs fabricated via GLAD can act as a buffer or bridging layer to promote charge transfer and structural integration. Vertical wurtzite-phase CdTe NaPAs with tailored optical properties were deposited on soda–lime glass substrates via glancing angle sputtering deposition (Figure 3h) [83]. A 100 nm-thick wurtzite CdTe interlayer, left intact after standard CdCl_2_ treatment, bridged the crystal structure gap between hexagonal CdS and zinc-blended CdTe, resulting in an approximately 0.9% increase in efficiency compared to planar sputtered CdTe/CdS cells. This approach may be extended to other thin-film solar cells, where lattice alignment can be significantly enhanced through interfacial engineering with appropriately matched interlayers, leading to improved photovoltaic performance [162,163].

### 4.2. Perovskite Solar Cells

Organic–inorganic hybrid metal halide perovskites and all-inorganic perovskites have emerged as leading candidates for next-generation photovoltaics due to their high light absorption, tunable bandgap, easier solution production process, and low cost [164,165]. The photovoltaic-active perovskites are typically made of an ABX_3_ perovskite structure, where A represents a monovalent organic or inorganic cation (such as methylammonium (MA^+^), formamidinium (FA^+^), or cesium (Cs^+^)), B is a divalent metal cation (commonly Pb^2+^ or Sn^2+^), and X represents a halide anion (such as I^−^, Br^−^, or Cl^−^) [166]. By tuning the A, B, or X-site ions, the perovskites can achieve varied defect tolerances and charge transport properties, contributing to their superior optoelectronic performance [167,168]. Current research focuses on improving PCE while ensuring operational stability, also critical for scalable commercialization of PSCs [169,170].

GLAD-fabricated nanostructures have been integrated into PSCs to enhance charge transport, mechanical flexibility, and device stability. Recent research has primarily focused on their application within ETLs, HTLs, and interface layer, where tailored NaPAs provide aligned pathways for efficient carrier extraction, reduced interfacial resistance, and improved uniformity of films [171].

#### 4.2.1. Electron Transporting Layers

**Figure 4:**
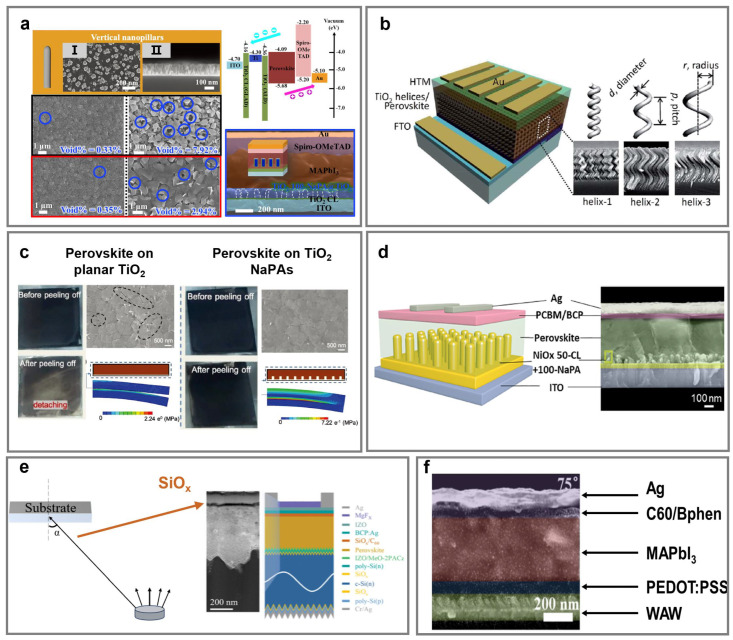
Photovoltaic applications of GLAD. (**a**) Top-view and cross-sectional SEM images of vertically aligned Ti nanopillar arrays (NaPAs) fabricated by GLAD and the perovskite films deposited on them with reduced void density, and the schematic device structure of the perovskite solar cell incorporating TiO_2_ NaPAs with the corresponding energy band diagram. (I), (II) show the top-view and cross-sectional SEM images of Ti NaPAs, while blue circles indicate voids in the MAPbI_3_ films. The void ratio increased to 7.59% for pristine TiO_2_ films but only 2.59% for Ti NaPA-based films after 50 days of aging, confirming improved interfacial stability. (**b**) Schematic of helical TiO_2_ NaPA-based perovskite solar cell and cross-section SEM images of three different helical TiO_2_ NaPAs, helix-1, helix-2, and helix-3, grown on silicon wafer. (**c**) Photographs and SEM images comparing planar TiO_2_ and vertical TiO_2_ NaPAs before and after mechanical testing. Left: Tape-peeling test images demonstrating enhanced interfacial adhesion with NaPAs. Right: SEM images and finite element simulations after 200 bending cycles, showing fewer cracks and lower stress concentration in perovskite films deposited on vertical NaPAs. Dashed lines indicate crack regions in the planar TiO_2_ sample. (**d**) Schematic and cross-sectional diagram of inverted perovskite solar cell with NiO*_x_* NaPAs as hole transporting layer. (**e**) Deposition illustration and cross-sectional SEM image of a two-terminal monolithic perovskite/silicon tandem solar cell with a glancing-angle-deposited SiO*_x_* interlayer. (**f**) Cross-sectional SEM image of an inverted perovskite solar cell employing a vertical WO_3_/Ag/WO_3_ NaPAs electrode deposited at a glancing angle of 75°. (**a**) Adapted with permission [54]. Copyright 2020, Wiley. (**b**) Adapted with permission [62]. Copyright 2014, Royal Society of Chemistry. (**c**) Adapted with permission [173]. Copyright 2020, Wiley. (**d**) Adapted with permission [84]. Copyright 2019, American Chemical Society. (**e**) Adapted with permission [81]. Copyright 2024, Wiley. (**f**) Adapted with permission [86]. Copyright 2018, American Chemical Society.

In traditional n-i-p PSCs, compact or mesoporous TiO_2_ layers are widely used as ETLs. However, these layers often require high-temperature sintering (>500 °C) and suffer from poor reproducibility [172]. To address the problem, a versatile alternative is provided by GLAD which enables precise control over nanostructure geometry and deposition conditions, allowing the development of ETLs compatible with low-temperature processing. It was noted that various titanium (Ti) nanostructures including tilted, vertical, helical, and zigzag NaPAs were successfully deposited via GLAD [54]. Among diverse Ti NaPAs, vertically columnar Ti NaPAs, which were grown on rigid ITO-coated glasses and flexible ITO-coated PET (Figure 4a) and then were subsequently coated with a conformal TiO_2_ layer using ALD, form the Ti 100-NaPA@TiO_2_ core@shell structure. TiO_2_ conformal layers enabled uniform coverage along high-aspect-ratio NaPAs, minimizing interfacial voids and enhancing electron extraction. Thus, the maximum PCE of the PSCs exhibited an increase of approximately 19%. Highly suppressed voids at the perovskite grain boundaries were observed in Ti@TiO_2_ films, contributing to improved film quality and device performance.

Park et al. deposited three helical TiO_2_ NaPAs (namely helix-1, helix-2, helix-3) with different pitch and radius (Figure 4b) using GLAD [62]. The high porosity of the helical TiO_2_ NaPAs (~63%), estimated via ellipsometry analysis of refractive index, ensures sufficient contact to facilitate efficient perovskite precursor contact during the two-step dipping process. Finally, the helix-1 with the smallest pitch and highest number of helical turns at a given film thickness offered the largest specific surface area. Improved crystallinity and interfacial contact, enabled by the helical geometry, were directly reflected in the enhanced PCE.

GLAD-fabricated TiO_2_ NaPAs have also been directly deposited on flexible substrates at room temperature, demonstrating the potential for low-temperature processing. The flexible PSCs incorporating these vertically columnar TiO_2_ NaPAs achieved a PCE of 13.3% and retained high photovoltaic performance after 500 instances of bending at a 1.5 cm radius. To assess the mechanical stability of the perovskite/ETL interface, scotch tape peeling tests and finite element analysis simulations (FEA) were conducted [173]. PSCs employing planar TiO_2_ exhibited evident delamination of the perovskite film, whereas those based on TiO_2_ NaPAs retained intact morphology after peeling (Figure 4c). Corresponding FEA simulations further confirmed that the stress at the interface of TiO_2_ NaPAs was significantly higher (7.22 MPa) than that of planar structures (2.24 MPa), indicating stronger interfacial adhesion due to the mechanical interlocking effect of the vertical NaPAs. This robust adhesion enhances mechanical flexibility and long-term durability in flexible PSCs.

Tin oxides have attracted lots of attention as alternative ETLs to TiO_2_ for flexible and stable PSCs. For example, Huang et al. reported on large-area (1 cm^2^) flexible PSCs incorporating the columnar SnO_2_ NaPAs, which achieved a maximum PCE of 14.9% [79]. The devices exhibited only 10% degradation after 800 h of ambient storage and approximately 20% efficiency loss after 400 bending cycles, while avoiding oxygen vacancy defects.

In all-inorganic PSCs, the engineering of ETLs remains a critical challenge due to energy band mismatch. With GLAD-fabricated TiO_2_ NaPAs acting as the effective ETL, the devices incorporating vertical TiO_2_ NaPAs achieved a PCE of 11.35% compared to 10.04% for planar devices and retained 60% of their efficiency after thermal aging at 50 °C for 10 days [63]. Despite these improvements, the overall device performance remained limited by low perovskite crystallinity, which continues to be a critical focus for further optimization toward commercialization [174].

#### 4.2.2. Hole Transporting Layer and Interface Engineering

While GLAD-engineered ETLs have shown promise in n-i-p devices, their integration into inverted p-i-n devices has further expanded their versatility across various functional layers like hole transporting layers, interface layers, and composite electrodes.

Hole extraction efficiency, energy-level alignment, light management, and mechanical flexibility are still critical in inverted PSCs [175,176,177]. For instance, vertically columnar NiO*_x_* NaPAs fabricated via GLAD on flexible substrates, functioning as HTLs, exhibited enhanced optical transmittance and reduced exciton recombination [84]. Devices integrated with NiO*_x_* NaPAs achieved PCEs of 20% on rigid substrates and 17% on flexible substrates and maintained crack-free morphology after 500 bending cycles, consistent with finite element simulations (Figure 4d). This robust fabrication demonstrates potential for large-area, high-efficiency flexible PSCs.

GLAD has been utilized for interfacial engineering in inverted tandem PSCs. Recently, via glancing-angle thermal evaporation, an ultrathin SiO*_x_* layer was deposited directly onto the perovskite surface without damage [81]. Condensed SiO*_x_* thin layers effectively passivated uncoordinated Pb^2+^ defects and introduced an n/n^+^ homojunction that enhanced field-effect passivation, suppressing non-radiative recombination while improving electron extraction [178]. Incorporating this interlayer into a two-terminal monolithic perovskite/tunnel oxide passivating contact silicon tandem solar cell yielded a stabilized PCE of 30.2%, one of the highest PCEs reported to date for this configuration (Figure 4e). Moreover, the dense, uniform SiO*_x_* acts as an internal encapsulation layer, contributing to excellent operational stability under both light (ISOS-L-1) and thermal (ISOS-D-2I) stress conditions [179].

Liu et al. developed multilayer transparent electrodes composed of WO_3_/Ag/WO_3_ (WAW), where the top WO_3_ was deposited at a 75° glancing angle to form vertically aligned NaPAs [86]. The vertical WO_3_ NaPAs enhanced light scattering and reduced reflection, thereby improving light absorption in PSCs. The central Ag layer provides electrical conductivity, and the bottom WO_3_ layer improves adhesion and releases mechanical strain during bending. Flexible PSCs incorporating this WAW structure retained 90.97% of their initial PCE after 1000 bending cycles at 1.3% strain, outperforming planar WAW electrodes which retained only 78.39% (Figure 4f).

## 5. Photodetection

For decades, photodetectors based on semiconductors including silicon carbide, silicon, indium gallium arsenide and germanium have exhibited excellent photosensitivity but have also been constrained by several factors, such as high production costs, high-temperature processing requirements, flexible substrate incompatibility, and limited spectral range for efficient operation. GLAD-enabled oxide photodetectors span UV detection based on wide-bandgap oxides; visible-to-near-infrared (NIR) detection, utilizing heterostructures and plasmonic effects; and flexible photodetectors designed for wearable applications. To comprehensively evaluate the photodetection performance of these nanostructures, a series of key figures of merit (including photoresponsivity, specific detectivity, noise equivalent power, and response speed) are characterized to reflect the efficiency, sensitivity, and speed of photodetection [180].

Vertically aligned, high-aspect-ratio NaPAs fabricated by GLAD significantly enhance the optical interaction volume of photodetectors, leading to superior light trapping and more effective photon absorption across a broad spectral range [181,182]. Moreover, these nanostructures also provide direct and continuous pathways for charge transport, which facilitates rapid photogenerated carrier separation and reduces recombination losses, particularly in devices where space-charge regions or interfacial barriers are critical to performance [183,184,185]. In addition, the GLAD technique offers precise control over the composition and spatial distribution of functional NPs on NaPA surfaces, enabling the formation of hybrid junctions, such as Schottky, Ohmic, or p-n interfaces that further boost internal electric fields, enhance hot carrier injection, and extend the operational spectral range of the device [186,187,188].

Representative examples are presented to demonstrate the diverse functions enabled by GLAD-fabricated nanostructures, explaining how tailored NaPAs not only support broadband light harvesting and efficient carrier extraction but also contribute to mechanical robustness under operational stress. To sum up, these studies demonstrate the effectiveness of GLAD-based structural engineering in advancing photodetector performance across wide spectral ranges and device configurations.

### 5.1. UV Photodetection Using NaPAs Made from Wide-Bandgap Oxide Semiconductors

Wide-bandgap oxides such as TiO_2_, In_2_O_3_, CeO_2_, Ga_2_O_3_, Gd_2_O_3_, and ZrO_2_ are attractive for UV photodetection due to their large bandgaps, thermal stability, and chemical stability [189,190]. When structured into NaPAs via GLAD, they exhibit enhanced UV light absorption, primarily owing to their tailored morphologies that increase the effective optical path length and surface interaction.

**Figure 5:**
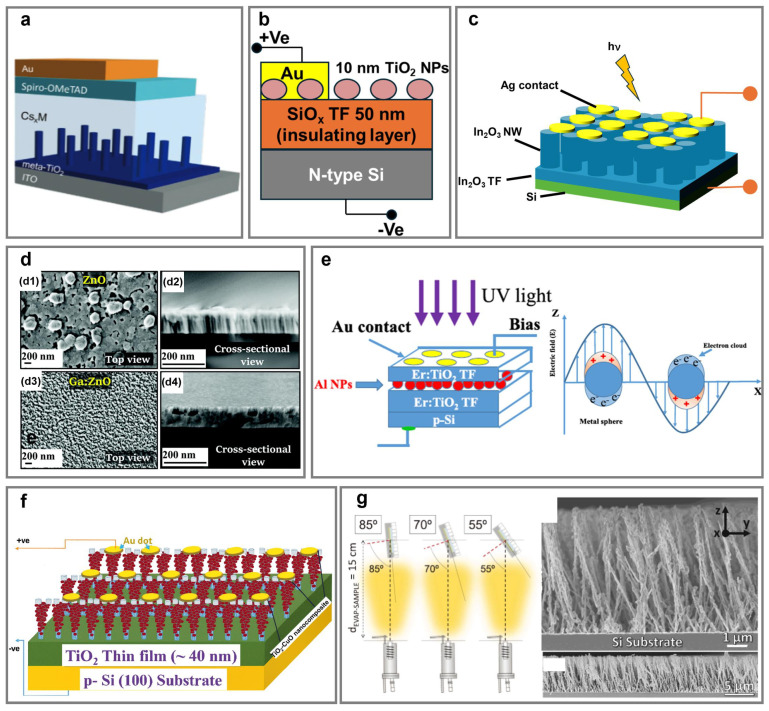
Photodetection applications of GLAD. (**a**) Schematic of a perovskite photodetector integrated with TiO_2_ nanopillar arrays (NaPAs) between Cs*_x_*M and TiO_2_. (**b**) Schematic of TiO_2_ nanoparticle-based photodetector. (**c**) Schematic of the Ag/vertical In_2_O_3_ NaPAs/In_2_O_3_ thin film/p-Si photodetector structure. (**d**) SEM images of ZnO and Ga-doped ZnO NaPAs: (**d1**,**d2**) top-view and cross-sectional views of pristine ZnO, and (**d3**,**d4**) those of Ga:ZnO, showing enhanced structural uniformity upon Ga doping. (**e**) Schematic of Au/Er:TiO_2_/Al nanoparticles/Er:TiO_2_/p-Si device and resonant oscillation in conduction electrons of metal nanoparticles under the oscillating electric field of the incident light. (**f**) Schematic of the vertical CuO-TiO_2_ NaPAs and TiO_2_ thin-film heterojunction photodetector under illumination at zero bias. (**g**) Schematic of PbI_2_ nanowalls deposited at different glancing angles and corresponding cross-sectional SEM images with different scale bars. (**a**) Adapted with permission [64]. Copyright 2020, Wiley. (**b**) Adapted with permission [65]. Copyright 2014, IEEE. (**c**) Adapted with permission [85]. Copyright 2021, IEEE. (**d**) Adapted with permission [91], Copyright 2020, Royal Society of Chemistry. (**e**) Adapted with permission [66], Copyright 2023, Elsevier. (**f**) Adapted with permission [204]. Copyright 2024, Elsevier. (**g**) Adapted with permission [96]. Copyright 2022, Wiley.

TiO_2_ is the most commonly utilized wide-bandgap semiconductor for UV detection and electron transport, but its conventional processing typically requires high-temperature annealing (>450 °C) to achieve desirable crystallinity and conductivity, which limits its integration with flexible or temperature-sensitive substrates [191,192]. In contrast, GLAD enables the direct deposition of vertically aligned TiO_2_ NaPAs at room temperature, offering a low-temperature alternative for device fabrication. When using them as ETLs in self-powered perovskite photodetectors, vertically aligned TiO_2_ NaPAs resulted in a ∼25% increase in photocurrent density compared to mesoporous TiO_2_ or blocking layers (Figure 5a) [64]. The improvement is due to suppressed carrier recombination and enhanced electron extraction at the TiO_2_/Cs*_x_*M interfaces. Similarly, GLAD-deposited TiO_2_ NPs on thermally oxidized silicon substrates (SiO*_x_*/Si) exhibited strong photoluminescence at 378 nm, corresponding to a 3.28 eV bandgap (Figure 5b) [65]. Corresponding Au/TiO_2_ Schottky photodetectors indicated a responsivity of 0.05 A/W and external quantum efficiency (EQE) of 16% at 378 nm. However, they also achieved a relatively high ideality factor of 11.4 that shows substantial non-ideal behavior, likely arising from interface defects or trap states, which induce unfavorable ion migration or recombination [193].

Chinnamuthu et al. introduced flexible UV photodetectors that were fabricated using TiO_2_@Ge NaPAs grown on Ti foils via a two-step process [56]. Vertically aligned TiO_2_ NaPAs were initially synthesized hydrothermally, followed by Ge shell deposition via glancing angle magnetron sputtering. The TiO_2_@Ge NaPAs formed a type-II band alignment where adjacent domains of semiconductors had band gaps that formed a staggered alignment at the heterojunction, which promoted a spatial separation of the electron and hole following photoexcitation. Excellent UV responsivity (0.123 A/W at 640 nm), high on/off ratio (~13), and fast response times were characterized in this case. Importantly, the TiO_2_@Ge photodetectors delivered excellent mechanical flexibility, retaining over 93% of their initial photocurrent after 1000 cycles at a bending radius of 5 mm.

GLAD has also been extended to other wide-bandgap oxide semiconductors for enhanced UV and deep-UV photodetection, with each exhibiting unique benefits. In_2_O_3_ is distinguished by both its wide bandgap and high electron mobility, making it highly suitable for deep-UV detection. The photodetector with the columnar In_2_O_3_ NaPAs exhibited excellent photoresponse with a rise at 0.6519 s and decay at 0.51 s under UV (340 nm) illumination (Figure 5c) [85]. The vertically columnar Ga_2_O_3_ NaPAs deposited on p-type Si wafers via GLAD showed dual bandgaps (3.2 eV and 4.5 eV) due to anisotropic growth and oxygen vacancies [87]. The Au/Ga_2_O_3_/p-Si photodetectors possessed high responsivity (2.29 A/W at 300 nm, 6.54 A/W at 400 nm) and low dark current (~7.55 × 10^−9^ A), maintaining stable performance across a wide temperature range (300–523 K), favorable for circuit integration in deep-UV applications. Gd_2_O_3_ with a wide bandgap (~5.4 eV), high thermal stability, and low phonon energy has been used to develop a self-powered UV photodetector. Vertical Gd_2_O_3_ NaPAs, decorated with Ag NPs and annealed at 700 °C, exhibited high responsivity and detectivity under 325 nm illumination, which remained for 100 cycles [88]. Raman et al. explored the effect of annealing temperature on ZrO_2_ NPs fabricated via GLAD for UV photodetection [89]. The as-deposited films were believed to form an amorphous morphology with limited carrier transport but annealing at 800 °C significantly improved crystallinity and reduced intrinsic defect states. When the ZrO_2_ NPs were integrated into a photodetector, the annealed photodetectors demonstrated a responsivity of 33 A/W and detectivity of 1.96 × 10^9^ Jones. The study highlights how GLAD-enabled morphological control, when combined with thermal treatment, can be leveraged to optimize charge transport and boost UV photodetection efficiency in wide-bandgap metal oxides.

Modification of oxide NaPAs with plasmonic NPs is favorable to enhance UV photodetection. Vertically columnar CeO_2_ NaPAs decorated with Ag NPs demonstrated a responsivity of 18.74 A/W under 370 nm illumination [90]. Embedded Ag NPs induce LSPR to strengthen local electric fields and promote hot electron injections, which accelerates charge separation and suppresses recombination. Effective UV photodetection also benefits from the high electron mobility of CeO_2_ [194].

ZnO emerges as a versatile UV photodetection material owing to its facile elemental doping and substrate compatibility. Vertically columnar ZnO NaPAs fabricated via pulsed laser-induced GLAD on various substrates, including quartz, sapphire (Al_2_O_3_) (001), and MgO (100), displayed substrate-dependent performance [195]. ZnO NaPAs grown on sapphire (001) resulted in the highest photosensitivity (182%) and the fastest response, attributed to their reduced defect density [196]. Furthermore, Ga-doped ZnO NaPAs showed a significantly amplified sensitivity of 210%, compared to 59% for undoped ZnO (Figure 5d) [91]. A transient photocurrent at 10 K confirmed their excellent thermal stability.

Interestingly, amorphous materials like silica also enable UV photodetection when sculpted into columnar NaPAs. Vertically columnar silica NaPAs achieved six-fold higher photosensitivity compared to the silica planar films [82]. Effective hole trapping at the silica/indium Schottky interface reduces the depletion width and barrier height, thereby facilitating more efficient carrier collection [197,198].

### 5.2. Broadband (UV-Visible-Near-Infrared) Photodetection Using Heterostructure Photodetectors

Broadband visible-NIR photodetection has been realized using GLAD-fabricated heterostructures combined with wide and narrow bandgap semiconductors, as well as with strategically embedded plasmonic NPs. Heterostructures fabricated via GLAD not only broaden the absorption spectrum but also introduce LSPR-induced light absorption and charge separation. For instance, multilayer TiO_2_/Ag/TiO_2_ NaPAs with each layer deposited at 80° enabled self-powered UV-visible photodetection and suggested substantial responsivity under both UV and visible illumination [199]. Within the device, the bottom and top TiO_2_ layers act as a dielectric layer, while the intermediate Ag layer provides LSPR to enhance optical absorption in the active region [200]. Another design involves a vertically stacked multilayer composed of Au/Er-doped TiO_2_/Al NPs/Er-doped TiO_2_ on p-Si substrates for NIR photodetection (Figure 5e) [66]. The Al NPs, positioned between two Er-doped TiO_2_ layers and thermally evaporated at 85°, introduce an LSPR effect to significantly increase photocurrent density under NIR (808 nm) illumination [201].

The core@shell design has been utilized to create self-powered broadband photodetectors [202,203]. For instance, Co_3_O_4_@TiO_2_ NaPAs form an effective p-n junction at the core–shell interface, enabling efficient charge separation and extending photoresponse into the visible region (~500 nm), while maintaining high responsivity and structural stability [67]. Additionally, CuO@TiO_2_ NaPAs have been explored to engineer light-responsive interfaces that facilitate efficient carrier dynamics (Figure 5f) [204]. On the other hand, vertically columnar Cu_2_O@TiO_2_ NaPA photodetectors demonstrated rapid and reversible responses, with rise and fall times of 0.153 s and 0.164 s at 750 nm [94]. Hierarchical core–shell structures provide a large surface area for oxygen adsorption and efficient energy band alignment [205]. Upon illumination, photogenerated holes desorb the oxygen species, narrowing the depletion region and enhancing the local electric field, thereby improving charge collection [206]. Heterojunction design has proven effective in achieving self-powered photodetection with high responsivity, fast response times, and excellent mechanical flexibility spanning the UV to NIR spectrum.

In addition, quaternary chalcogenides have also been explored for broadband photodetection [207]. Vertically aligned CuI_0.7_Ga_0.3_Se_2_ (CIGS) NaPAs were fabricated via RF magnetron sputtering with a quaternary CIGS target. The CIGS NaPAs exhibited strong light absorption of up to 90% of incident light, from UV to visible wavelengths (up to 550 nm), compared to ~70% by the compact layers [95]. The morphology-driven absorption enhancement, along with the intrinsic tunability of the CIGS bandgap via doped Ga modulation highlights the promise of GLAD-deposited CIGS NaPAs for low-cost, high-efficiency broadband photodetectors.

Mechanical flexibility has become increasingly vital for wearable photodetector applications. Hierarchical ZnO NaPAs were prepared on ultrathin honeycomb-structured Si membranes, achieving broadband photodetection across 365 to 1100 nm [196]. The devices exhibited rise/fall times of 11 ms/12 ms, respectively, and maintained over 90% of their initial performance after 10,000 bending cycles. The enhanced flexibility originates from both the low-modulus Si membrane and the vertical ZnO NaPA geometry, which together adjust the strain and maintain performance under repeated deformation.

### 5.3. Emerging Polarization-Sensitive Photodetection

Polarization-sensitive photodetection based on anisotropic materials has been extensively investigated in recent years. A structural anisotropic strategy induced via GLAD has emerged as an effective method to achieve polarization detectivity. Barranco et al. employed a two-step thermal evaporation process to fabricate anisotropic methylammonium lead iodide (MAPbI_3_) nanowalls using GLAD. Initially, PbI_2_ was deposited at a glancing angle, followed by CH_3_NH_3_I at a normal incidence angle, resulting in well-aligned MAPbI_3_ nanowalls at room temperature [96]. As depicted in Figure 5g, owing to their anisotropic alignment and symmetry breaking, these nanowalls exhibit intrinsic chirality. Notably, the MAPbI_3_ nanowalls showed enhanced responsivity under CPL, distinguished by their preferential crystalline orientation along the (110) plane, which can be finely tuned through the deposition angle and nanowall density [208,209]. Leveraging geometrically controlled nanowalls enables CPL sensitivity without chiral organic molecules, inspiring novel design strategies for polarization-sensitive optoelectronic devices.

GLAD-fabricated photodetectors based on diverse materials offer a series of key advantages, including wavelength selective responsivity, tunable bandgap characteristics, enhanced light–matter interaction, and compatibility with flexible optoelectronics. Continued research into defect passivation, carrier mobility enhancement, and scalable integration will be crucial for fully realizing the potential of GLAD-enabled photodetectors, particularly in emerging fields like polarization-sensitive and broadband photodetection.

## 6. Photocatalysis

Photocatalysis represents another application area for GLAD-fabricated nanostructures, particularly in solar-driven water splitting, pollutant degradation, and CO_2_ reduction. However, practical photocatalytic performance is often restricted by insufficient light utilization, rapid electron–hole recombination, and slow surface kinetics [210]. By engineering highly controlled nanostructures such as zigzag, helical, and vertically columnar NaPAs composed of noble metals, metal sulfides, and various semiconductors, it is possible to create nanostructures with large surface areas, enhanced light absorption, and tunable density, all of which are critical for maximizing photocatalytic efficiency.

TiO_2_-based NaPAs have been widely studied as a representative GLAD-fabricated photocatalyst due to their structural uniformity and reliable performance [55]. To further enhance light utilization and reaction efficiency, recent studies have explored a broad range of materials and structural engineering strategies. These include the use of visible-light-responsive oxides and sulfides, heterostructure and core@shell designs for interfacial charge transfer, as well as elemental doping and defect modulation for tailored bandgap alignment.

### 6.1. Wide-Bandgap NaPAs for UV-Driven Photocatalysis

**Figure 6:**
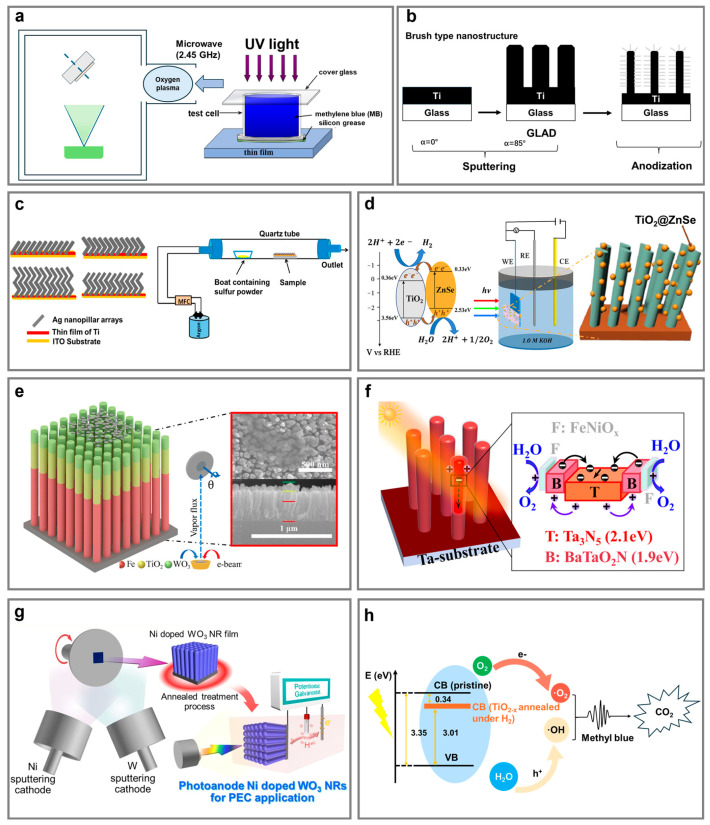
Photocatalytic applications of GLAD. (**a**) Schematic of oxygen-ion-assisted glancing-angle-deposited TiO_2_ nanopillar arrays (NaPAs) and methylene blue photoinduced degradation device. (**b**) Schematic of Ti-based brush-type nanostructures using GLAD-fabricated NaPAs followed by anodization. (**c**) Schematic diagram GLAD fabrication of different arms of zigzag Ag NaPAs and sulfidation using two-zone furnace. (**d**) Band diagram of TiO_2_ NaPAs@ZnSe NP heterostructure for efficient oxygen evolution reaction. The orange dashed box magnifies the TiO_2_@ZnSe heterostructure, showing ZnSe nanoparticles uniformly coated on TiO_2_ nanorods. (**e**) Schematic and SEM images of electron-beam-evaporated ternary heterostructure comprising vertical Fe_2_O_3_/TiO_2_/WO_3_ NaPAs coupled with Ti_3_C_2_T_x_ MXene nanosheet. (**f**) Schematic of Ta_3_N_5_@BaTaO_2_N NaPA photoanode for solar water splitting and corresponding band energy diagram with efficient charge separation process. Vertically aligned Ta_3_N_5_@BaTaO_2_N and FeNiO*_x_* cocatalyst enable electrons migrate through the Ta_3_N_5_ to the Ta substrate, while holes transfer to the BaTaO_2_N/FeNiO*_x_* surface to drive efficient water oxidation. (**g**) Schematic of Ni-doped WO_3_ NaPA photoanode fabrication via co-sputtering and annealing for photo-driven water splitting. (**h**) Schematic illustration of the band structure modulation and photocatalytic mechanism of TiO*_2-x_* NaPAs annealed in hydrogen for efficient methyl blue degradation. The orange arrow represents the conduction-band electron transfer that reduces adsorbed O_2_ to radicals (·O_2_). The light-yellow arrow denotes the valence-band hole oxidation process, where H_2_O molecules are oxidized to hydroxyl radicals (·OH). (**a**) Adapted with permission [68], Copyright 2022, AIP Advances. (**b**) Adapted with permission [55]. Copyright 2014, IOP Publishing. (**c**) Adapted with permission [53]. Copyright 2021, Elsevier. (**d**) Adapted with permission [72]. Copyright 2023, Springer Nature. (**e**) Adapted with permission [58]. Copyright 2024, Springer Nature. (**f**) Adapted with permission [57]. Copyright 2020, American Chemical Society. (**g**) Adapted with permission [59]. Copyright 2022, Elsevier. (**h**) Schematic illustration inspired by [71]. Copyright 2017, Elsevier.

TiO_2_ remains one of promising photocatalysts due to its chemical stability, non-toxicity, and favorable bandgap for UV-driven reactions [211]. For instance, vertically columnar TiO_2_ NaPAs, fabricated via GLAD combined with oxygen-ion-assisted reactive evaporation and annealing at 650 °C, exhibited excellent superhydrophilicity with a water contact angle below 5° after one hour of UV irradiation (Figure 6a) [68]. The TiO_2_ NaPAs achieved a methylene blue degradation rate approximately 14-fold higher than that of compact TiO_2_ thin films, attributed to an increase in surface area and the formation of an anatase phase with superior charge mobility [212]. The TiO_2_ NaPAs also demonstrated unique photoinduced self-regeneration. Brett et al. deposited vertically columnar TiO_2_ NaPAs on interdigitated electrodes, producing humidity sensors with capacitance changes exceeding 1000 fold between 2% and 95% relative humidity and sub-second response times [69]. Over time, device performance degraded due to surface contamination and reduced hydrophilicity, but after 40 h of UV exposure, performance was restored and even enhanced due to photocatalytic degradation of surface contaminants, emphasizing the self-regenerative potential of GLAD-fabricated TiO_2_ NaPAs.

Pihosh et al. fabricated vertically aligned Ti NaPAs via GLAD which were subsequently anodized to generate hierarchical nanotube and brush-type nanostructures (Figure 6b) [55]. The brush-type nanostructures possessed increased specific surface areas and intrinsic AR properties, both of which are critical for maximizing light absorbance and utilization. Photocatalytic degradation of isopropanol under UV irradiation revealed that the nanotube and brush-type samples outperformed the compact TiO_2_ films and the columnar TiO_2_ NaPAs, whereby the nanotubes enabled complete decomposition of isopropanol.

ZnO represents the alternatives due to its wide bandgap and intrinsic advantages in defect engineering and doping flexibility for visible–light–driven photocatalysis. ZnO NPs fabricated by GLAD-pulsed laser deposition followed by annealing at 550 °C exhibited superior photoelectrochemical water-splitting performance with a photocurrent of 142.5 μA/cm^2^ and a photon-to-hydrogen efficiency of 0.6%, owing to a high surface-to-volume ratio and efficient charge transport [92]. Moreover, the demonstrated hydrophilicity and self-regenerating behavior under UV exposure arising from modulated oxygen vacancy further suggests their suitability for stable, long-lifetime photocatalytic systems.

### 6.2. Narrow-Bandgap NaPAs for Visible Light-Driven Photocatalysis

Chalcogenide materials have also attracted significant interest due to their narrow bandgaps and high absorption coefficients for visible light-driven photocatalysis. Singh et al. produced zigzag Ag_2_S NaPAs through a two-step process, where Ag NaPAs were firstly deposited using GLAD and subsequently converted into Ag_2_S via controlled sulfurization treatment (Figure 6c) [53]. By tuning the number of arms in the zigzag, the four-arm zigzag Ag_2_S NaPAs showed the highest photocurrent density of 3.05 mA/cm^2^, attributed to enhanced light absorption and minimized resistance at the semiconductor electrolyte interface.

Vertical columnar Cu_2_O NaPAs prepared by glancing angle sputter deposition demonstrated a 25% increase in photocurrent density in H^+^/H_2_ reactions compared to compact Cu_2_O films, which was linked to the higher surface area and a preferred [111] crystal orientation [93,213]. The donor carrier density was elevated from 3.1 × 10^21^ cm^−3^ to 2.5 × 10^22^ cm^−3^. Additionally, the space-charge region width decreased from 43 nm to 16 nm, strengthening the internal electric field and facilitating efficient migration of photogenerated electrons and holes to the active sites.

### 6.3. Interface Engineering of NaPAs to Enhance Photocatalysis

Interface engineering through the formation of heterojunctions and core@shell nanostructures has been proven for essentially enhancing photocatalytic performance. The strategies promote directional charge transfer, suppress electron–hole recombination, and optimize energy band alignment at the interface [214]. Heterojunctions are typically formed by integrating semiconductors with staggered band structures, enabling spatial charge separation, whereas core@shell design promotes radial charge transport and improves surface redox kinetics by shortening carrier diffusion lengths and increasing available reaction sites.

#### 6.3.1. Heterojunction Structures

Improving the visible-light response of wide-bandgap semiconductors (e.g., TiO_2_, ZnO, WO_3_) has been a central focus in photocatalytic research, as these materials are typically only active under UV illumination [215]. A widely adopted strategy involves coupling them with narrow-bandgap semiconductors such as Fe_2_O_3_, Cu_2_O, CuO, CdSe, and CdTe to form heterojunctions that extend absorption into the visible region [216].

Proper band alignment between different semiconductors promotes directional charge transfer across the interfaces, enabling efficient separation of excitons. For instance, vertically columnar TiO_2_ NaPAs were modified with Ti_3_C_2_T_x_ MXene nanosheets via spin-coating, yielding a nearly 40% increase in photocurrent density compared to TiO_2_ compact layers [217]. The incorporation of MXenes facilitates charge carrier separation and suppresses exciton recombination. Moreover, the composites also exhibited excellent long-term photocatalytic stability under continuous illumination, suggesting strong potential for solar-driven water splitting and other photocatalytic applications. Wei et al. reported that ZnSe/TiO_2_ heterojunction NaPAs attained enhanced photocatalytic activity under visible illumination (Figure 6d) [72]. The ZnSe/TiO_2_ NaPAs achieved a photocurrent density 2.3-times higher than that of the pristine TiO_2_ NaPAs and 45.35% degradation of methyl orange within 8 h, compared to only 27.36% by TiO_2_ alone. Furthermore, the heterojunction structure enabled efficient O_2_ reduction and •OH generation via surface-trapped electrons and holes, resulting in complete mineralization of dye molecules.

To further optimize multilayer charge dynamics, as shown in Figure 6e, a ternary heterostructure composed of vertically columnar Fe_2_O_3_/TiO_2_/WO_3_ NaPAs integrated with Ti_3_C_2_T_x_ MXene nanosheets was constructed for efficient photoelectrochemical water splitting [58]. The resulting device achieved a remarkably high photocurrent density of 1.09 mA/cm^2^ under simulated sunlight, representing a 36-times enhancement over the pristine Fe_2_O_3_ NaPAs. Synergistic multi-step electron transfer enabled by the serial n-n and Schottky heterojunctions facilitates efficient charge separation and transport.

#### 6.3.2. Core@shell Structures

Core@shell nanostructures offer an advanced strategy to maximize interfacial contact, promote directional charge separation, and ultimately improve photocatalytic efficiency [218,219]. These systems typically benefit from type-II band alignment, where the conduction band minimum (CBM) and valence band maximum (VBM) of the two semiconductors are staggered. This configuration enables electron migration from a material with a higher CBM to one with a lower CBM, while holes simultaneously move in the opposite direction, thereby facilitating effective charge separation and suppressing carrier recombination [220].

Specifically, α-Fe_2_O_3_@TiO_2_ NaPAs were engineered to maximize interfacial contact and facilitate charge separation for enhanced photocatalytic activity [70]. Post-annealing treatment at 450 °C enabled the crystallization of α-Fe_2_O_3_ and anatase TiO_2_ to form a type-II heterojunction that drove electron transfer from Fe_2_O_3_ to TiO_2_ while confining holes in Fe_2_O_3_, thus suppressing charge recombination. The optimized α-Fe_2_O_3_@TiO_2_ NaPAs exhibited a methylene blue degradation rate of 0.151 h^−1^ under visible illumination, outperforming both pristine Fe_2_O_3_ NaPAs (0.121 h^−1^) and TiO_2_ NaPAs (0.034 h^−1^). Meanwhile, the α-Fe_2_O_3_@TiO_2_ NaPAs also enabled the photoconversion of carbon dioxide into carbon monoxide, hydrogen, methane, and methanol after only a few hours of exposure under ambient sunlight, surpassing the activity of both pure vertical anatase TiO_2_ and α-Fe_2_O_3_ NaPAs. These results further confirm the advantages of the GLAD fabricated core–shell design in enhancing photocatalytic performance.

The vertically columnar Ta_3_N_5_@BaTaO_2_N NaPAs combined with dip-coated FeNiO*_x_* catalysts achieved high photocurrent density and maintained Faradaic efficiencies of approximately 96% (Figure 6f) [57]. Likewise, vertically columnar WO_3_@BiVO_4_ NaPAs fabricated by GLAD-DC magnetron sputtering outperformed pristine columnar WO_3_ NaPAs (1.04 mA/cm^2^) and columnar BiVO_4_ (1.24 mA/cm^2^) NaPAs and highlighted 2.24 mA/cm^2^ with high photo-stability [221]. This enhanced performance is attributed to the favorable type-II band alignment, which promotes charge separation, as confirmed by the suppressed PL emission and reduced interfacial resistance observed in EIS measurements.

Sulfide-based core@shell nanostructures have also been developed to exploit similar principles of improved charge separation and generation of reactive species. It is notable that the Fe_3_O_4_ NaPAs@CdS NPs were synthesized via GLAD followed by thermal oxidization in air, yielding optimized photocatalytic activity for the degradation of methylene blue under visible illumination [222]. The optimal composite attained a degradation efficiency of 95% within 60 min, outperforming both CdS NPs (74%) and Fe_3_O_4_ NaPAs (27%). The effective charge separation across the CdS/Fe_3_O_4_ interface enhanced the generation of reactive oxygen species such as •O_2_^−^ and •OH radicals [223].

#### 6.3.3. Doping and Defect-Engineering of NaPAs for Bandgap Modulation

Elemental doping of either non-metals (such as sulfur, fluorine, or nitrogen) or noble metals (e.g., silver, gold, platinum) tailors the electronic band structure and introduce mid-gap states that promote visible-light photocatalysis [224,225].

For example, the vertically columnar Ni-doped WO_3_ NaPAs were prepared using GLAD followed by post-annealing to introduce phase transformation and crystallinity (Figure 6g) [59]. Co-sputter with Ni led to a photocurrent density of 1.1 mA/cm^2^ at 1.23 V vs. RHE under AM 1.5G illumination, nearly three times higher than the undoped WO_3_ NaPAs (0.38 mA/cm^2^). The synergistic effects of Ni-induced conductivity, improved crystallinity, and the porous vertical alignment of NaPAs enhanced the photoelectrochemical performance. The results were further supported by EIS measurements, which revealed a significant reduction in charge-transfer resistance upon Ni doping.

Defect engineering has also proven effective for modulating TiO_2_ band structure and improving its visible-light response. Hydrogen annealing of vertically columnar TiO_2_ NaPAs generated oxygen-deficient TiO*_2-x_* phases enriched with Ti^3+^ species and oxygen vacancies (Figure 6h) [71]. This treatment narrowed the optical bandgap from 3.35 eV to 3.01 eV, boosting absorption in the visible range and reducing charge carrier recombination. The modified films exhibited a significantly enhanced photocatalytic performance, as evidenced by a methylene blue degradation rate nearly twice that of the untreated compact TiO_2_ films under AM 1.5G solar illumination.

In addition to the intrinsic advantages of GLAD-fabricated nanostructures, recent advances have incorporated interfacial engineering, elemental doping, and post annealing treatment to improve photocatalytic and photoelectrochemical performance. Combined with the precise morphological control provided by GLAD, these strategies synergistically promote charge separation, enable tailored band alignments, and optimize surface and interface properties, thus expanding the functional versatility of the GLAD-derived nanostructures in photocatalysis.

## 7. Light Emitting Diodes

Through precise control over nanostructure orientation and density, GLAD-fabricated NaPAs can simultaneously function as optical AR coatings and thermal management interlayers in LED devices. This approach eliminates the need for lithography or substrate patterning, streamlining fabrication and enabling multifunctional integration across advanced LED systems.

**Figure 7:**
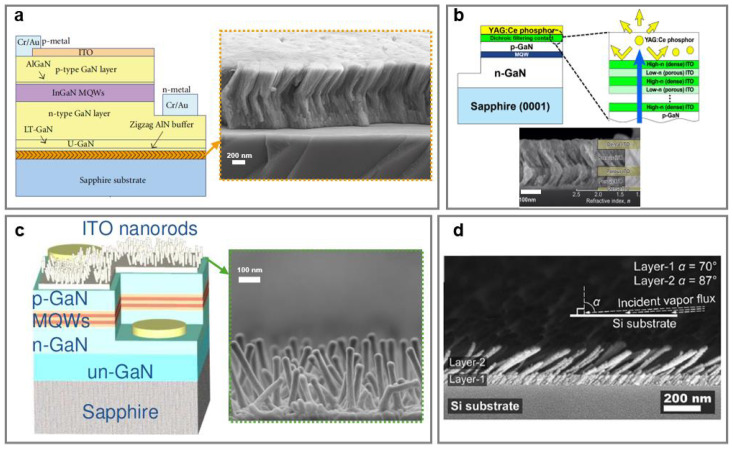
Applications of GLAD in light-emitting diodes. (**a**) Schematic of GaN-based LED incorporating a GLAD zigzag AlN nanopillar array (NaPA) buffer layer and corresponding cross-section SEM image of zigzag AIN NaPAs. (**b**) Schematic of a phosphor-converted dichroic white LED with dichroic-filtering contacts (DFC) consisting of an alternating stack of dense ITO and tilted ITO NaPAs and SEM image of the DFC. (**c**) Schematic of fabricated GaN based LEDs with titled ITO NaPAs. (**d**) Cross-sectional SEM image of the two-layer titled Al_2_O_3_ NaPAs deposited on a Si. (**a**) Adapted with permission [97]. Copyright 2012, Wiley. (**b**) Adapted with permission [75]. Copyright 2013, Royal Society of Chemistry. (**c**) Adapted with permission [76]. Copyright IOP Science. (**d**) Adapted with permission [98]. Copyright AIP Advances.

Zigzag and helical aluminum nitride (AlN) NaPAs have been employed as buffer layers for GaN-based LEDs, leading to enhanced light extraction and thermal stress tolerance. Specifically, zigzag AlN NaPAs achieved a 28.6% increase in light output power at an injection current of 20 mA compared to the planar buffer layers, primarily attributed to their increased surface roughness and enhanced light-scattering (Figure 7a) [97]. GLAD-derived zigzag NaPAs as anisotropic buffer layers optimized the optoelectronic interfaces without relying on patterned substrates. GaInN-based LEDs with graded-refractive-index (GRIN) AR coatings, fabricated by GLAD, have been demonstrated as an effective strategy to reduce Fresnel reflection and enhance light extraction. Kim et al. proposed a six-layer GRIN comprising zigzag NaPAs made from ITO with individually tuned refractive indices (ranging from dense ITO (high-n) to ITO NaPAs (low-n) deposited by GLAD) achieved a 24.3% enhancement in light-extraction efficiency, compared to the conventional dense ITO.

Similarly, when applied in phosphor-converted GaN-based white LEDs, which typically utilize a blue-emitting chip combined with a yellow phosphor layer, overall conversion efficiency is constrained by the backward emission of yellow fluorescence toward the chip, where it goes through partial reabsorption and introduces optical losses. To overcome this limitation, Kim et al. developed ITO-based dichroic-filtering contacts (DFCs) consisting of compact ITO films and tilted ITO NaPAs via GLAD. DFCs serve as both blue-transmitting, yellow-reflecting optical filters and low-resistance ohmic contacts to p-type GaN (Figure 7b) [75]. Incorporating 3- and 5-layer DFCs into GaInN/GaN multiple quantum well (MQW) LEDs resulted in an improvement of phosphor conversion efficiency to 9.8% and 17.7%, respectively, while maintaining efficient carrier injection. This strategy effectively mitigates backward fluorescence loss and provides a scalable, lithography-free approach to enhance light-extraction in phosphor-converted white LEDs.

The vertically aligned ITO NaPAs functioning as a broadband transparent conductive layer (TCL) achieved high transmittance (>90%) from 450 to 900 nm (Figure 7c) [76]. In the packaged InGaN/GaN LEDs, the ITO NaPAs resulted in a 35.1% enhancement in light output power at a drive current of 350 mA, compared to the devices with compact TCLs. Electromagnetic simulations using finite-difference time-domain methods revealed that increasing the nanorod thickness could further improve the extraction enhancement factor.

For deep-UV AlGaN-based LEDs, where Fresnel reflection at the air interface severely limits photon outcoupling, tilted Al_2_O_3_ NaPAs featuring controlled density hava been employed as AR coatings (Figure 7d) [98]. A dual layer coating with 172 nm thickness obtained negligible (<2%) extinction, demonstrating a viable approach for deep-UV optical coating applications.

In a visable III-V AlGaInP LED, vertically aligned antimony(Sb)-doped tin oxide (ATO) NaPAs were uniformly deposited via glancing-angle sputtering onto a p-type GaP window layer with a large area (1 × 1 mm^2^) functioning as a low-refractive-index (n ≈ 1.8) AR coating [99]. The vertical ATO NaPAs significantly suppressed internal Fresnel reflection and improved light outcoupling, resulting in a 26% increase in optical power at 350 mA. The reduced photon reabsorption in the MQWs minimized thermal effects, leading to a smaller electroluminescence peak redshift under high injection current, holding the potential for GLAD AR coatings to improve large-area LED efficiency.

The applications of GLAD in LEDs are largely restricted to optical and interfacial layers due to the strict epitaxial growth, doping precision, and lattice matching requirements in III-V semiconductor active layers, which are not compatible with physical vapor deposition techniques. Nevertheless, the unique structural and optical advantages of GLAD-engineered NaPAs suggest substantial potential for advancing circularly polarized organic LEDs (CP-OLEDs) which emerge as a rising star for 3D displays. Achieving CP-OLED with both a high electroluminescence asymmetry factor and high external quantum efficiency remains a challenge, primarily due to the difficult balance between chiroptical activity and charge transport. Helical or tilted NaPAs fabricated via GLAD can serve either as chiral-emitting layers or as integrated DFCs, enhancing the high degree of circular polarization and light extraction. Their anisotropic morphology, tunable chiroptical activity, and compatibility with diverse materials are likely to make them a versatile method for realizing high-efficiency, structurally controlled CP-OLEDs.

## 8. Summary and Perspectives

This review comprehensively summarized the recent advances in the research and applications of GLAD for optoelectronic devices [74,97,152]. GLAD stands out as a highly versatile and controllable bottom-up fabrication method that enables the formation of diverse nanostructures, including vertically aligned NaPAs, zigzag NaPAs, helical NaPAs, nanotrees, and metallic NPs without requiring lithographic patterning or templating techniques [226,227]. These sculpted nanostructures exhibit a high degree of tunability in morphology, composition, crystallinity, and anisotropy, allowing fine manipulation of optical, electrical, mechanical, and chiroptical properties. Hence, GLAD has been successfully applied across a broad range of optoelectronic devices, including photovoltaics, photodetectors, photocatalysts, and LEDs.

**Opportunities, Challenges, and Pathways for Scaling GLAD-Enabled Optoelectronics.** Importantly, the function of GLAD-fabricated NaPAs is closely determined by their nanostructural design, which enables them to address fundamental challenges in photovoltaics, photodetectors, photocatalysts, and LEDs. These optoelectronics leverage the structural versatility of GLAD, where vertical columnar NaPAs can enhance charge carrier transport and light absorptions in solar cells, zigzag NaPAs and branched nanotrees increase interfacial areas for photochemical reactions, and helical NaPAs with chiral morphology enable polarization-sensitive detection. In LEDs, anisotropic zigzag NaPAs and tilted NaPAs help reduce optical losses, thermal accumulation, and act as reflection barriers. Despite the differing operational principles of these devices, whether photon-driven or electrically injected, they all share common critical processes, i.e., efficient light absorption or injection, charge separation, and interface carrier dynamics. By tailoring the morphology and composition of NaPAs, GLAD engineering facilitates synergetic improvements in light-matter interaction processes across diverse devices.

Despite these advantages, several challenges currently limit broader applications of GLAD in optoelectronics. First, the technique is inherently constrained by its reliance on high-vacuum electron-beam evaporation, which restricts the material library to oxides and metals with high melting points, while excluding many functional organics, polymers, and halide perovskites that have attracted much attention to emerging devices recently. Second, scaling from centimeter-scale to wafer-scale deposition is inherently difficult because the directional vapor flux is not perfectly uniform over large areas. Variations in effective incidence angle and local deposition rate across a substrate lead to spatial nonuniformity in NaPAs’ tilt angles, densities, and heights. These nonuniform effects are further influenced by the source-to-substrate distance, spatial variations in vapor fluxes across a substrate, constraints in coordinating multiple axis substrate motion, and temperature nonuniformity during prolonged deposition. Third, device integration on non-planar or soft substrates is fundamentally constrained by the directional, non-conformal nature of GLAD. For instance, on textured wafers with facets, V-pits, and trenches, changes in surface orientation cause regions to intercept the vapor at different angles, make nanopillars grow in different directions, and often stop growth in bridge valleys. On randomly textured or microlens-type surfaces, curvature spreads the incidence angles to induce nonuniform growth and mixed morphologies that could not be repeatably formed. Nanoimprinted gratings and meta-surface reliefs with steep sidewalls and narrow trenches receive much less flux in recessed regions than exposed ridges, leaving voids and incomplete sidewall coverage. Porous membranes and fibers (e.g., AAO, porous Si, Ni foam) have relatively rough surfaces that interrupt film growth, leading to the formation of discontinuous NaPAs and poor electrical contact. In these cases, GLAD does not yield conformal, ordered NaPAs, and the outcome is site-selective growth with gaps and irregular interfaces, which undermines optical or electrical coupling in optoelectronic integration.

**Figure 8:**
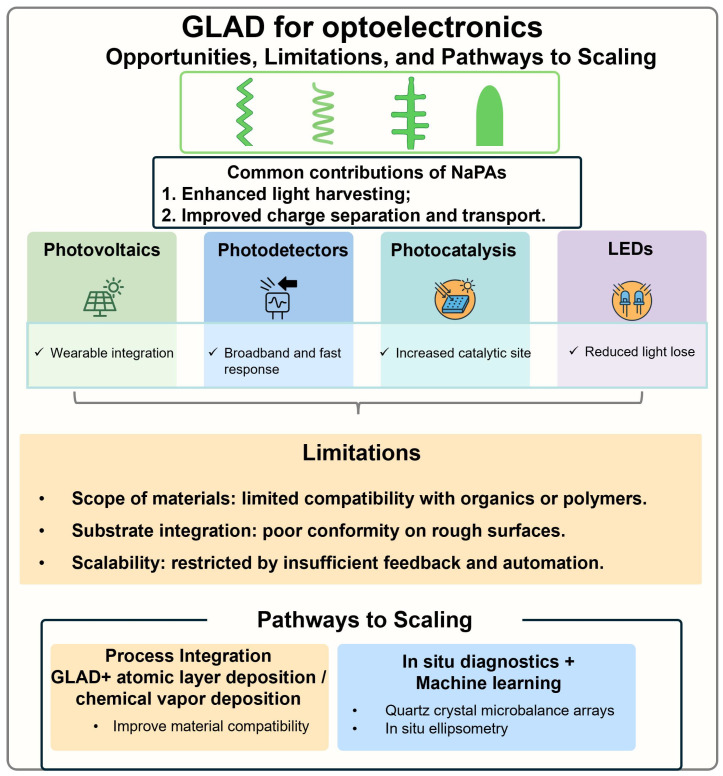
Schematic roadmap illustration of the opportunities, limitations, and scaling pathways of GLAD-enabled optoelectronics.

Opportunities, challenges, and pathways towards potential scaling-up are summarized in Figure 8. To advance GLAD from the laboratory toward manufacturable optoelectronics, progress is needed on several fronts. Hybrid process integration should pair GLAD with conformal deposition (e.g., ALD, low-temperature CVD, or solution-coating methods) to broaden the accessible material scope; with this, GLAD imparts anisotropy and optical enhancement, while the conformal coating ensures coverage and interface stability. Moreover, strict control of the evaporation source and substrate motion, together with quantitative in situ metrology, is essential. Real-time monitoring of deposition using spatially distributed quartz crystal microbalance sensors and spectroscopic ellipsometry mapping can provide in situ data on thickness, growth rate, and optical anisotropy. Coupling these signals with model-based or machine learning-assisted feedback offers a viable route toward adaptive optimization, ensuring uniform morphology and consistent nanostructure evolution across large-area substrates. Finally, integration with textured or non-planar substrates requires robust and automated alignment protocols, including calibrated step-and-tilt deposition strategies that account for local surface orientation and enable conformal nanostructure growth across topographically varied regions. These alignments can be guided by in situ diagnostic feedback to dynamically adjust substrate positioning during deposition, thereby precisely preserving the functional anisotropy essential for device performance.

**Extension of the scope of GLAD-sculpted fabrication.** Despite the notable advances of GLAD-enabled nanostructures in diverse optoelectronic devices, several challenges hinder their broader application, particularly in scaling up for industrial production and integrating with existing fabrication processes. Currently, GLAD processes primarily rely on electron-beam evaporation, which is constrained to materials with high melting or sublimation points and low vapor pressures under vacuum [228,229]. This severely limits the material to mostly oxides and metals, thereby excluding organic semiconductors, low-temperature polymers, or other functional compounds with poor thermal stability. To address this, developments are expected to focus on GLAD-compatible vapor delivery systems, including aerosol-assisted and plasma-enhanced approaches, which enable deposition at lower temperatures using volatile or reactive precursors [230,231,232]. These emerging strategies may enable the integration of GLAD with solution-processable materials, such as metal halide perovskites and organic–inorganic halide semiconductors. Meanwhile, coupling GLAD with atomic layer deposition, low-temperature chemical vapor deposition, or solution-based processes offers a promising pathway toward hybrid fabrication. For instance, inorganic GLAD-fabricated NaPA templates can act as scaffolds for subsequent conformal coating of organic or low-melting-point functional materials, thereby creating vertically aligned hybrid nanostructures with expanded material versatility [233,234].

**Integration of NaPAs with two-dimensional (2D) materialsand topological insulators.** Given the intrinsic link between nanostructure geometry and material functionality, advancing GLAD-based device applications will also rely on strategic integration of emerging materials. Since the discovery of mono-layered graphene, 2D nanosheets and layered materials have attracted tremendous attention for applications ranging from photocatalysis and energy storage to electrocatalysis, magnetoresistance, and sensing. A particularly promising direction involves integrating 2D materials with GLAD-fabricated nanostructures. Two-dimensional semiconductors such as transition metal dichalcogenides (TMDs, e.g., MoSe_2_, MoS_2_, WS_2_) possess layer-dependent tunable bandgaps, high carrier mobilities, and strong light–matter interactions [235,236]. Although direct growth of TMDs via GLAD remains challenging due to their high-vacuum instability and thermal sensitivity [237,238], GLAD-fabricated vertical or helical NaPAs can act as structural scaffolds or integration templates onto which 2D materials are subsequently introduced using complementary techniques such as CVD or ALD. For instance, black phosphorus, with its strong in-plane anisotropy can be incorporated onto GLAD templates to broaden the spectral response range of photodetectors, while hexagonal boron nitride deposited by CVD onto NaPAs forms atomically smooth dielectric interfaces that suppress charge traps and minimize carrier scattering.

Furthermore, GLAD-enabled fabrication of chiral or anisotropic topological insulator nanostructures (e.g., Bi_2_Se_3_, Bi_2_Te_3_, Sb_2_Te_3_) creates prospects for coupling the angular momentum of light with spin-polarized electronic states [239]. Topological insulators exhibit insulating bulk and spin-momentum-locked surface states protected by time-reversal symmetry [240]. Combined with strong spin–orbit coupling enabling robust spin-polarized charge transport without the need for external magnetic fields, their surface states enable efficient generation and manipulation of spin currents, paving the way for low-power, high-speed, spin-based devices. However, achieving controlled symmetric breaking over spin-polarized transport remains a critical challenge. GLAD-fabricated helical NaPAs offer stronger symmetry breaking and enhanced spin-polarized photocurrents primarily through the circular photogalvanic effect, which directly links the polarized light to charge transport [241,242]. Moreover, their strong spin–orbit coupling supports spin Hall type responses, further expanding the mechanisms available for spin current control. While direct GLAD of topological insulators remains challenging due to volatility and stoichiometric sensitivity, GLAD-derived helical NaPAs can serve as versatile scaffolds or templates for topological insulators synthesized through complementary methods such as MBE, PLD, ALD. The hybrid nanostructures not only provide a pathway toward spin-optoelectronic devices, exemplified by circularly polarized photodetectors, but also offer promise for next-generation spintronic devices, including ultrafast spin logic switches.

**In situ monitoring and automation of GLAD.** Improvements in process monitoring and automation are critical for achieving reproducible and scalable GLAD fabrication. The integration of in situ diagnostic tools (e.g., specroscopic ellipsometry, X-ray photoelectron spectroscopy (XPS), or reflection high-energy electron diffraction (RHEED)) [243,244,245] provides real-time insights into the dynamic evolution of nanostructures during deposition. Specifically, spectroscopic ellipsometry enables continuous tracking of density and columnar tilt angle, thereby correlating anisotropic optical properties (e.g., birefringence, dichroism) with deposition parameters such as incidence angle (α) and substrate rotation (φ).

In GLAD, where glancing incidence and shadowing effects drive competitive nucleation, RHEED provides a powerful means to monitor crystal growth along glancing directions and detect morphological transitions from columnar to dense structures. The information given by in situ RHEED effectively helps optimize grain orientation and surface state characteristics for optoelectronics [246,247]. Likewise, in situ XPS enables real-time probing of how reactive gas atmospheres (e.g., O_2_, H_2_) influence oxidation states, defect densities, and chemical bonding, which guides optimization of trap density and carrier lifetime.

Through in situ monitoring, closed-loop process control enhanced by machine learning (ML) algorithms is looking forward to improving reproducibility, structural precision, and manufacturing throughput. Supervised ML models including support vector machines [248], decision trees [249], and random forests [250] have been applied to predict film morphology, thickness uniformity, and surface roughness based on real-time process parameters [251,252]. Unsupervised learning models, such as *k*-means clustering and principal component analysis are effective for anomaly detection and classifying growth modes from RHEED data [245]. More recently, deep learning models, particularly convolutional neural networks, have been integrated with real-time imaging tools (e.g., RHEED or SEM images) to extract crystalline characteristics and dynamically guide nanostructure evolution [253,254]. By correlating deposition conditions with structural characteristics of NaPAs, including diameter, density, and helical pitch and linking these to device-level metrics (e.g., absorption, carrier lifetime, and polarization sensitivity), ML enables predictive optimization of GLAD-grown nanostructures. These innovations are essential to scale GLAD toward wafer-level, defect-tolerant manufacturing of advanced optoelectronics.

In summary, GLAD holds strong potential in constructing structurally and functionally diverse nanostructures for optoelectronic applications. However, its broader development is limited by material compatibility and process integration. To overcome these challenges, future work requires both material innovation and process engineering. Combining GLAD fabrication with emerging materials such as 2D materials and topological insulators paves the way for expanding functionality into new optoelectronics. As for the deposition process, integrating GLAD with low-temperature deposition techniques, in situ diagnostic tools, and machine learning-assisted control systems will be essential for improving deposition precision, structural reproducibility, and scalability. With continued progress in both material integration and intelligent process optimization, GLAD is expected to evolve from a laboratory-scale technique to a robust nanomanufacturing technology for next-generation optoelectronics.

## Figures and Tables

**Table 1:** Representative materials deposited using GLAD with diverse evaporation processes, acting as optoelectronic functional layers.

Material	Deposition Method	Refs.
**Metals**		
Ag	Electron beam evaporation	[52,53]
Ti	Electron beam evaporation, Sputtering	[54,55]
Ge	Electron beam evaporation	[56]
Ta	Sputtering	[57]
Fe	Electron beam evaporation	[58]
Ni	Sputtering	[59]
**Inorganic compounds**		
TiO_2_	Electron beam evaporation	[52,58,60,61,62,63,64,65,66,67,68,69,70,71,72]
ITO	Electron beam evaporation	[73,74,75,76,77]
SnO*_x_*	Sputtering, electron beam evaporation	[78,79,80]
SiO*_x_*	Electron beam evaporation	[81,82]
SiO_2_	Electron beam evaporation	[61]
CdTe	Thermal evaporation	[83]
NiO*_x_*	Electron beam evaporation	[84]
In_2_O_3_	Electron beam evaporation	[85]
WO_3_	Sputtering, thermal evaporation, electron beam evaporation	[58,59,86]
Ga_2_O_3_	Thermal evaporation	[87]
Gd_2_O_3_	Electron beam evaporation	[88]
ZrO_2_	Electron beam evaporation	[89]
CeO_2_	Electron beam evaporation	[90]
ZnO	Pulsed laser deposition, sputtering	[91,92]
Co_3_O_4_	Electron beam evaporation	[67]
Cu_2_O	Electron beam evaporation, sputtering	[93,94]
CIGS	Sputtering	[95]
PbI_2_	Thermal evaporation	[96]
Fe_2_O_3_	Electron beam evaporation	[70]
AlN	Vapor–liquid–solid	[97]
Al_2_O_3_	Electron beam evaporation	[98]
Antimony (Sb)-doped tin oxide	Sputtering	[99]
**Organic molecules**		
Copper phthalocyanine	Thermal evaporation	[100]
Chloroaluminum phthalocyanine	Thermal evaporation	[101]

## Data Availability

No primary research results, software, or code have been included, and no new data were generated or analyzed as part of this review.
